# The TNF-Alpha-238 Polymorphism and Cancer Risk: A Meta-Analysis

**DOI:** 10.1371/journal.pone.0022092

**Published:** 2011-07-19

**Authors:** Ping Zhou, Guo-Qiang Lv, Jian-Zhong Wang, Cheng-Wan Li, Liang-Feng Du, Chun Zhang, Jian-Ping Li

**Affiliations:** 1 Department of General Surgery, The Third Affiliated Hospital to Nantong University, Wuxi, China; 2 Institute of Integrated Traditional Chinese and Western Medicine, The Third Affiliated Hospital to Nantong University, Wuxi, China; 3 Department of Intensive Care Unite, The Third Affiliated Hospital to Nantong University, Wuxi, China; University of Swansea, United Kingdom

## Abstract

**Background and Objectives:**

Tumor necrosis factor-α (TNF-α) plays a very important role in the development and progress of cancer. Some TNF-α polymorphisms have been confirmed to increase cancer risks; however, the association between TNF-α-238 polymorphism and cancers remains controversial and ambiguous. The aim of this study is to explore a more precise estimation of its relationship with cancer using meta-analysis.

**Methods:**

Electronic searches of several databases were conducted for all publications on the association between this variant and cancer through March 2011. Odds ratios (OR) with 95% confidence intervals (95% CI) were used to access the strength of this association in the random-effect model.

**Results:**

Thirty four studies with 34,679 cancer patients and 41,186 healthy controls were included. This meta-analysis showed no significant association between TNF-α-238 polymorphism and cancers (AA+GA vs GG: OR = 1.09, 95%CI = 0.88–1.34). In Caucasian and Asian subgroups, OR values (95% CI) were 1.14 (0.91–1.43) and 0.97 (0.58–1.61), respectively. In the subgroups of cancer type, no significant association was detected. The sensitivity analysis further strengthened the validity of these negative associations. No publication bias was observed in this study.

**Conclusions:**

No significant association was found between the TNF-α-238 polymorphism and the risk for cancer.

## Introduction

Tumor necrosis factor-α (TNF-α) is the most important pro-inflammatory cytokine involved in the growth, differentiation, cellular function and survival of many cells. It is produced by diverse kinds of cells, such as macrophages, neutrophils, fibroblasts, keratinocytes, NK cells, T and B cells, and tumor cells [1]. TNF-α has been reported to play an important role in the pathogenesis of cancer [2].

As transcription of TNF-α is regulated under genetic control, recent studies [3]–[5] have shown that its promotor polymorphisms at −238 (rs361525), −308 (rs1800629), −857 (rs1799724), and −1031 (rs1799964) positions could regulate TNF-α production. TNF-α-308 polymorphism has been confirmed as a risk factor for a range of cancers by meta-analysis, such as breast, gastric and hepatocellular cancers [6]–[8]. Jang et al [9] reported that TNF-α-238 polymorphism might play an apparently protective role against cancers. And many studies have focused on the relation of this polymorphism to different types of cancer [4], [9]–[41]. However, an apparent discrepancy existed in the results. For the currently published studies only refer to a modest sample size and unified ethnicity, each of them might not achieve a reliable conclusion. Thus, we conducted this meta-analysis to combine the available studies [42].

### Publication search

Electronic databases (PubMed, EMBase, Cochrane Central Register of Controlled Trials and ISI Web of Science) were searched for all publications on the association between TNF-**α**-238 polymorphism and cancer through March 2011. The keywords were as follows: cancer/carcinoma, polymorphism/variant/genotype/SNP, and tumor necrosis factor/TNF-**α**. All the references of retrieved articles were also included as additional studies in this study. All the studies must meet the following criterias: (1) case-control study; (2) the outcome had to be cancer; and (3) at least two comparison groups (cancer group vs. control group).

### Data extraction

This meta-analysis included a total of 34 articles on TNF-**α**-238 and cancer. Two authors extracted the data independently and in duplicate. Items of the author's last name, year of publication, country of origin, source of the study population, genotypes and numbers of cases and controls and TNF-**α** genotyping method were extracted. The results were compared, disagreements were discussed, and consensus was reached. MOOSE Checklist and flow chart for the studies were shown as Checklist S1 and Figure S1.

### Statistical analysis

Review manager 5.0 was used to perform meta-analysis for TNF-**α**-238 polymorphisms (AA+GA versus GG genotype). The crude odds ratios (OR) and 95% CI were estimated for each study in a fixed- or random-effect model Heterogeneity among studies were examined with *I^2^* statistic interpreted as the proportion of total variation contributed by between-study variation. If there was a statistical difference in terms of heterogeneity (*P*<0.05), a random-effect model was selected to pool the data. A fixed-effect model, otherwise, was employed. Relative influence of each study on the pooled estimate was assessed by omitting one study at a time for sensitivity analysis. Funnel plots were used to evaluate publication bias. All *P*-values were two-tailed.

## Results

In this article, the association of TNF-α-238, −308 with cancer risk was investigated using meta-analysis in a range of populations. A total of 34 studies were identified to evaluate the relationship between TNF-**α**-238 polymorphism and risk for cancer. The detailed characteristics of the studies were shown in Table S1. 34,679 cancer patients and 41,186 healthy controls were included in this study. There were 9 studies for gastric cancer, 3 studies for breast cancer, 2 studies for lung cancer, 4 studies for hepatocellular carcinoma, 2 studies for myeloma, 2 studies for cervical cancer, 2 studies for oral cancer, 2 studies for prostate cancer, 2 studies for lymphoma and 5 studies for other 5 different cancers. Among those 34 studies, there were 16 Caucasus and 18 Asian studies, respectively.

**Figure 1:**
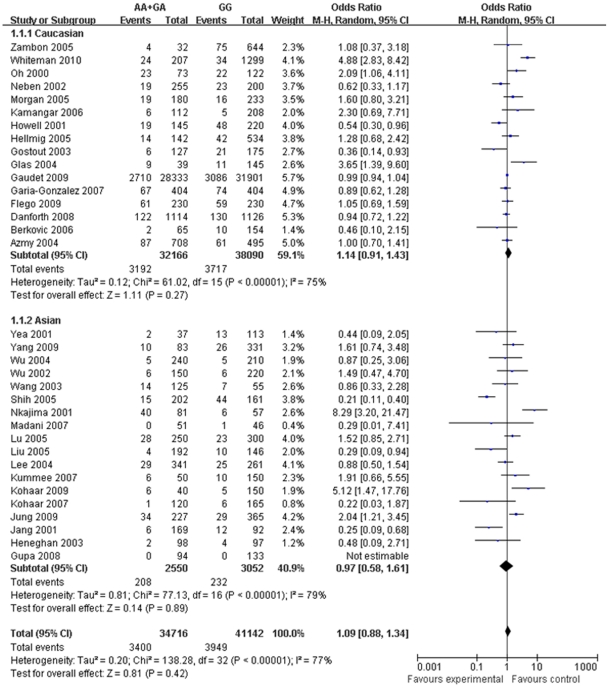
ORs and 95% confidence interval (CI) of cancer according to TNF-α-238 polymorphism in 34 studies using random-effect model. AA+GA, AA and GA genotypes of TNF-α-238 polymorphism; GG, GG genotype of TNF-α-238 polymorphism; ORs, odds ratios.

As shown in Table 1, the OR for cancers (95% CI) in overall studies with AA+AG vs GG of TNF-α-238 polymorphism was 1.09 (0.88–134, *P* = 0.42) (Table. 1). In Caucasian and Asian populations, ORs (95% CI) were 1.14 (0.91–1.43) and 0.97 (0.58–1.61), respectively. When stratified by the cancer type, the OR (95% CI) for gastric cancer was 1.22 (0.88–1.70) and for breast cancer being 1.13 (0.77–1.67); and similarly, values were 1.44 (0.82–2.52) and 0.99 (0.66–1.50) for hepatocellular carcinoma and other cancers. The forest plots of the meta-analysis were shown in Fig. 1.

**Table 1:** Results of pooled ORs in the meta-analysis.

Group	N	Case/control	OR (95% CI)	*P*
Overall	34	34679/41186	1.09(0.88,1.34)	0.42
Cancer type				
Gastric	9	1447/2633	1.22(0.88,1.70)	0.23
Breast	3	28443/29463	1.13(0.77,1.67)	0.52
Hepatocellular	4	500/667	1.44(0.82,2.52)	0.21
Others	18	4289/8423	0.99(0.66,1.50)	0.97
Ethnicity				
Caucasian	16	32165/38090	1.14(0.91,1.43)	0.27
Asian	18	2514/3096	0.97(0.58,1.61)	0.89

Furthermore, according to the analysis, we found that there was evidence of heterogeneity in the association between TNF-α-238 polymorphism and risk for cancers among the overall 34 and stratified studies: *I* ^2^ = 77% in overall populations, *I* ^2^ = 75% in Caucasus populations and *I* ^2^ = 79% in Asian populations. A random-effect model was employed in the ORs calculation. To further strengthen our findings, we conducted the sensitivity analysis. In the sensitivity analysis, there was little modification of the estimates after exclusion of individual study, with pooled ORs ranging from 1.02 to 1.13 for TNF-α-238 polymorphism.

**Figure 2:**
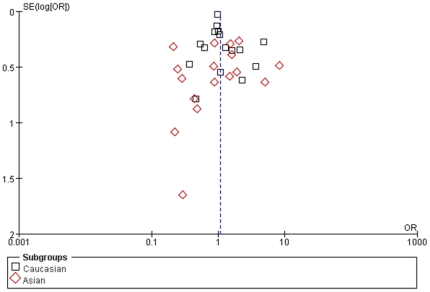
Funnel plots analysis to detect publication bias. Each point represents an independent study for the indicated association.

The shape of the funnel plots was prone to be symmetrical, suggesting that there was no evidence of publication bias among the studies (Fig. 2). The results of this meta-analysis leaded to our conclusion that no significant association was manifested between TNF-α-238 polymorphism and the risk for cancers.

## Discussion

As inflammation has been assumed as a key factor involving in the pathogenesis of cancer, TNF-α, the most crucial inflammatory cytokine, has been implicated in both the development and progression through pathways of “the NF-κB and AP-1 transcription factor complexes activation” in experimental and human cancer studies [2], [9]. Because A allele of TNF-α at −238 in the promoter region was found to down-regulate gene expression [5], [22], studies on the relationship between this variant and cancers has been extensively investigated during recent decades [4], [9]–[41]. However, results from these studies were ambiguous. To further provide insights into this debated subject, a meta-analysis is needed to achieve a more reliable and comprehensive conclusion.

To the best of our knowledge, we used meta-analysis to investigate the association between TNF-α-238 polymorphism and risk of cancers for the first time. With 34,679 cancer patients and 41,186 healthy controls included, this study had a greater power than all previous ones. We did not detect any significant association between TNF-α-238 polymorphism and cancer susceptibility in the overall population, with summary OR being 1.09 (0.88–1.34). Subgroup analyses stratified according to ethnicity and cancer type were also preformed, whilst with negative results acquired.

Because heterogeneity was found among the studies of overall and subgroups, random-effect model could be introduced to our analysis. Then, a sensitivity analysis was performed by removing one study for each time and re-running the model to determine the effect on the overall estimate. The estimates changed quite little, strengthening the results from this meta-analysis. Heterogeneity, however, still existed when each study was excluded in the sensitivity analysis, which indicated that population selection, cancer type and particular study were not the source of heterogeneity. Variability in frequency of this TNF-α-238 polymorphism among the populations or some unknown factors may be the source of heterogeneity. No publication bias was shown also suggesting this possible true result.

Several potential factors must be concerned with respects to the null association between TNF-α-238 polymorphism and cancers. First, cancer is a multi-factorial disease resulting from complex interactions between environmental and genetic factors [22]. It is possible that variants at this locus may have modest risks on cancers. Some environmental factors, however, may predominate in the development of cancer, such as living habits and exposure to carcinogens. Without considering these factors, it may lead to the failure to detect the role of TNF-α-238 polymorphism in cancer development. Second, some single-nucleotide polymorphisms of some cytokines, such as polymorphisms of interleukin-8-251, interleukin-10-819, transforming growth factor beta1-509 and TNF-α-308, may exert their complex and interacting functions with each other, which could affect the effects of TNF-α-238 polymorphism in the pathogenesis of cancer. Therefore, other polymorphisms as cancer risk factors should be taken into account to conclude a true effect. Third, this polymorphism might have different effects on some certain types of cancers. For example, interleukin-6-174CC genotype was a risk factor on bladder cancer while tends to be a protector on colorectal cancer and gastric caner [43]. However, the number of current studies of some particular cancer types is small, which means further investigations involving more cancer types are needed. Forth, TNF-α-308 polymorphism is significantly linked with higher occurrences of TNF-α-producing autoimmune Major Histocompatibility Complex (MHC) haplotype HLA-A1, B8, DR3, which was already confirmed as a risk factor for cancers [44]. However, it is still unknown whether TNF-α-238 polymorphism relates to MHC haplotype, which needs more investigation in the future. Thus, the lack of counting those factors above may affect the conclusions. This meta-analysis has pooled the available data from the case-control studies, which significantly increased statistical power. It cannot, however, overcome the above potentially critical factors.

In conclusion, no significant association was detected between TNF-α-238 polymorphism and cancers in this meta-analysis. Since the eligible case-control studies cannot provide a causal association, large well-designed cohort studies in the susceptibility of different types of cancer warrant to confirm this association in the future.

## Supporting Information

Figure S1 The flow chart of the included studies.
(DOC)

Table S1 (DOC)

Checklist S1 (DOC)
