# Supplementary material for: The TNF-Alpha-238 Polymorphism and Cancer Risk: A Meta-Analysis
Source: PLoS One. 2011 Jul 19;6(7):e22092. doi: 10.1371/journal.pone.0022092 (PMC3139602; doi:10.1371/journal.pone.0022092)
Supplement: Figure S1 — The flow chart of the included studies. (DOC) [file pone.0022092.s001.doc]

**Flow of Included Studies**

Potentially relevant studies identified and screened for retrieval (n=20820)

Studies excluded, without polymorphism reported (n=20406)

Studies retrieved for more detailed evaluation (n=414)

Potentially appropriate studies to be included in the meta-analysis (n=44)

Studies included in meta-analysis (n=34)

Studies excluded, with no *TNF* 238 genotypes (n=370)

Studies excluded, with no control group or cancer as outcome (n=10)
