# Supplementary material for: The TNF-Alpha-238 Polymorphism and Cancer Risk: A Meta-Analysis
Source: PLoS One. 2011 Jul 19;6(7):e22092. doi: 10.1371/journal.pone.0022092 (PMC3139602; doi:10.1371/journal.pone.0022092)
Supplement: Table S1 — (DOC) [file pone.0022092.s002.doc]

| Table S1. Characteristics of studies included in the meta-analysis | | | | | | | | |
| --- | --- | --- | --- | --- | --- | --- | --- | --- |
| Study | Year | Country | Ethnicity | Cancer type | Case/Control | Control/ SNP Frequency |  | Genotyping method |
| Azmy | 2004 | UK | Caucasian | Breast | 709/498 | GP/0.12 |  | Taqman |
| Berkovic | 2006 | Croatia | Caucasian | GEP-NETs | 65/184 | GP/0.03 |  | Taqman |
| Gostout | 2003 | US | Caucasian | Cervial | 127/175 | HP/0.05 |  | Sequencing |
| Gupta | 2008 | India | Asian | Oral | 94/133 | NR/0.00 |  | Nested-PCR |
| Heneghan | 2003 | China | Asian | Hepatocellular | 98/97 | GP/0.02 |  | PCP-RFLP |
| Howell | 2001 | UK | Caucasian | Malignant melanoma | 146/220 | GP/0.13 |  | PCR-SSOP |
| Jang | 2001 | Korea | Asian | Mix | 169/92 | GP/0.04 |  | PCR-RFLP |
| Kamangar | 2006 | Finland | Caucasian | Gastric | 112/208 | GP/0.05 |  | Taqman |
| Kohaar | 2009 | India | Asian | Breast | 40/150 | HP/0.15 |  | Sequencing |
| Kummee | 2007 | Thai | Asian | Hepatocellular | 50/150 | GP/0.12 |  | PCR-RFLP |
| Lee | 2004 | Korea | Asian | Gastric | 341/261 | GP/0.09 |  | Sequencing |
| Liu | 2005 | China | Asian | Oral | 192/146 | GP/0.02 |  | PCR-RFLP |
| Lu | 2005 | China | Asian | Gastric | 250/300 | NR/0.11 |  | DHPLC |
| Madani | 2007 | Iran | Asian | Colorectal | 51/46 | GP/0.00 |  | PCR-RFLP |
| Morgan | 2005 | UK | Caucasian | Myeloma | 181/233 | GP/0.11 |  | IHG |
| Nkajima | 2001 | Japan | Asian | Renal cell | 81/57 | GP/0.49 |  | Sequencing |
| Neben | 2002 | Germany | Caucasian | Myeloma | 255/200 | GP/0.07 |  | Sequencing |
| Oh | 2000 | US | Caucasian | Prostate | 73/122 | GP/0.32 |  | PCR-SSP |
| Shih | 2006 | China | Asian | Lung | 202/205 | GP/0.07 |  | Sequencing |
| Whiteman | 2010 | Australia | Caucasian | Esopageal | 208/1346 | GP/0.12 |  | Sequencing |
| Wu | 2002 | China | Asian | Gastric | 150/220 | GP/0.04 |  | Sequencing |
| Yang | 2009 | Korea | Asian | Gastric | 84/336 | GP/0.12 |  | SNaPshot |
| Yea | 2001 | Korea | Asian | Gastric | 37/113 | HP/0.05 |  | PCR-RFLP |
| Flego | 2009 | Croatia | Caucasian | Lung | 230/230 | GP/0.27 |  | PCR-RFLP |
| Kohaar | 2007 | India | Asian | Cervial | 120/165 | HP/0.01 |  | PCR-RFLP |
| Garia-Gonzalez | 2005 | Spain | Caucasian | Gastric | 404/404 | GP/0.17 |  | Taqman |
| Glas | 2004 | Germany | Caucasian | Gastric | 88/147 | GP/0.24 |  | Sequencing |
| Gaudet | 2009 | Mix | Caucasian | Breast | 30000/30000 | GP/0.10 |  | Mix |
| Jung | 2009 | Korea | Asian | Hepatocellular | 227/365 | GP/0.15 |  | Sequencing |
| Wang | 2003 | Japan | Asian | Hepatocellular | 125/55 | GP/0.11 |  | Sequencing |
| Hellmig | 2005 | Germany | Caucasian | Lymphoma | 144/534 | GP/0.10 |  | Taqman |
| Wu | 2004 | China | Asian | Lymphoma | 204/210 | HP/0.02 |  | Sequencing |
| Zambon | 2005 | Italy | Caucasian | Gastric | 32/644 | GP/0.13 |  | Sequencing |
| Danforth | 2008 | US | Caucasian | Prostate | 1159/1161 | GP/0.11 |  | Taqman |
| Gastroenteropancreatic neuroendocrine tumors (GEP-NETs);heteroduplex generator (IHG) analysis;PCR-based denaturing high-performance liquid chromatography (DHPLC);polymerase chain reaction-restriction fragment length polymorphism (PCR-RFLP) | | | | | | | | |
|  | | | | | | | | |
