# Supplementary material for: The TNF-Alpha-238 Polymorphism and Cancer Risk: A Meta-Analysis
Source: PLoS One. 2011 Jul 19;6(7):e22092. doi: 10.1371/journal.pone.0022092 (PMC3139602; doi:10.1371/journal.pone.0022092)
Supplement: Checklist S1 — (DOC) [file pone.0022092.s003.doc]

**MOOSE Checklist**

| **Criteria** | | **Brief description of how the criteria were handled in the meta-analysis** |
| --- | --- | --- |
| **Reporting of background should include** | |  |
| √ | Problem definition | Tumor necrosis factor alpha (TNF-α) is a crucial pro-inflammatory cytokine, which plays a role in the development of cancer. *TNF* 238 G/A polymorphism could lead to a changed *TNF* gene transcription. However, the association between this SNP and risk for cancer still remained unclear. The potential public health impact of *TNF* 238 G/A polymorphism on caner remains to be summarized quantitatively. |
| √ | Hypothesis statement | *TNF* 238 G/A polymorphism might influence the risk of cancer. |
| √ | Description of study outcomes | Cancer |
| √ | Type of exposure or intervention used | GA or AA of *TNF* 238 |
| √ | Type of study designs used | We included case-control studies, cross-sectional studies. |
| √ | Study population | We placed no restriction. |
| **Reporting of search strategy should include** | |  |
| √ | Qualifications of searchers | The credentials of the two investigators P.Z and CZ are indicated in the author list. |
| √ | Search strategy, including time period included in the synthesis and keywords | PubMed from 1965 –November 2010  EMBASE from 1974 –November 2010  Medline from 1965 –November 2010  diabetes, tumor necrosis factor and polymorphism or variant or genotype |
| √ | Databases and registries searched | PubMed, Medline and EMBASE |
| √ | Search software used, name and version, including special features | We did not employ any search software. EndNote was used to merge retrieved citations and eliminate duplications |
| √ | Use of hand searching | We hand-searched bibliographies of retrieved papers for additional references, |
| √ | List of citations located and those excluded, including justifications | Details of the literature search process are outlined in the flow chart. The citation list is available upon request |
| √ | Method of addressing articles published in languages other than English | We placed no restrictions on language; local scientists fluent in the original language of the article were contacted for translation |
| √ | Method of handling abstracts and unpublished studies | No unpublished studies were observed. |
| √ | Description of any contact with authors | We contacted authors who had conducted multivariate analysis with diabetes as a covariate, but had not reported relative risk for diabetes. |
| **Reporting of methods should include** | |  |
| √ | Description of relevance or appropriateness of studies assembled for assessing the hypothesis to be tested | Detailed inclusion and exclusion criteria were described in the methods section. |
| √ | Rationale for the selection and coding of data | Data extracted from each of the studies were relevant to the population characteristics, study design, exposure, outcome, and possible effect modifiers of the association. |
| √ | Assessment of confounding | No restricted for the analysis. Conducted sensitivity analyses by eliminating each study. |
| √ | Assessment of study quality, including blinding of quality assessors; stratification or regression on possible predictors of study results | The results of sensitivity analyses were very stable. |
| √ | Assessment of heterogeneity | Heterogeneity of the studies were explored within two types of study designs using Cochrane’s Q test of heterogeneity and I^2^ statistic that provides the relative amount of variance of the summary effect due to the between-study heterogeneity. |
| √ | Description of statistical methods in sufficient detail to be replicated | Description of methods of meta-analyses, sensitivity analyses and assessment of publication bias are detailed in the methods. |
| √ | Provision of appropriate tables and graphics | We included the terms used for database search, 1 flow chart, 2 summary table, 1 forest plot of all studies,1 funnel plots to examine publish bias. |
| **Reporting of results should include** | |  |
| √ | Graph summarizing individual study estimates and overall estimate | Figure 1 |
| √ | Table giving descriptive information for each study included | Table 1 |
| √ | Results of sensitivity testing | Not shown. |
| √ | Indication of statistical uncertainty of findings | 95% confidence intervals were presented with all summary estimates, *P* values and results of sensitivity analyses |
| **Reporting of discussion should include** | |  |
| √ | Quantitative assessment of bias | Sensitivity analyses indicate this non-significant association was stable. |
| √ | Justification for exclusion | We excluded studies that had used different exposure or outcome assessment for the comparison groups, or no control group. |
| √ | Assessment of quality of included studies | We discussed the results of the sensitivity analyses. |
| **Reporting of conclusions should include** | |  |
| √ | Consideration of alternative explanations for observed results | We discussed that potential unmeasured confounders such as other life style factors and cytokines may have caused non-significant results. |
| √ | Generalization of the conclusions | No significant association was detected between *TNF* 238 polymorphism and cancer. |
| √ | Guidelines for future research | We recommend future studies on the associations between *TNF* 238 polymorphism and specific cancers. |
| √ | Disclosure of funding source | No funding supported this study. |
